# Supplementary material for: Authentication of the Bilberry Extracts by an HPLC Fingerprint Method Combining Reference Standard Extracts
Source: Molecules. 2020 May 28;25(11):2514. doi: 10.3390/molecules25112514 (PMC7321295; doi:10.3390/molecules25112514)
Supplement: Supplementary file 1 [file molecules-25-02514-s001.pdf]

Authentication of the bilberry extracts by an HPLC fingerprint method combining reference standard extract

Bingbing Liu[1,2], Tiantian Hu[1], Weidong Yan[1,3 *]

1. Department of Chemistry, Zhejiang University, Hangzhou, 310027, China

2. State Key Laboratory of Environmental and Biological Analysis, Hong Kong Baptist University, Kowloon Tong, Kowloon, 999077, Hong Kong SAR, China

3. Zhejiang Skyherb Biotechnologies Co., Ltd., Anji, 313300, China

*Corresponding author

yanweidong@zju.edu.cn

Tel.: +86 571 8795 1430

Fax: +86 571 87951895

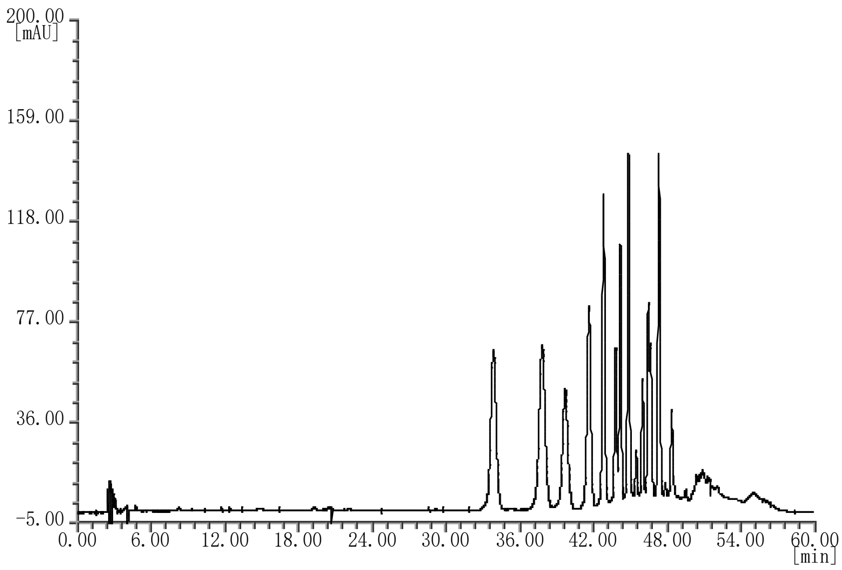

Figure. S1 Chromatogram under elution program 1.

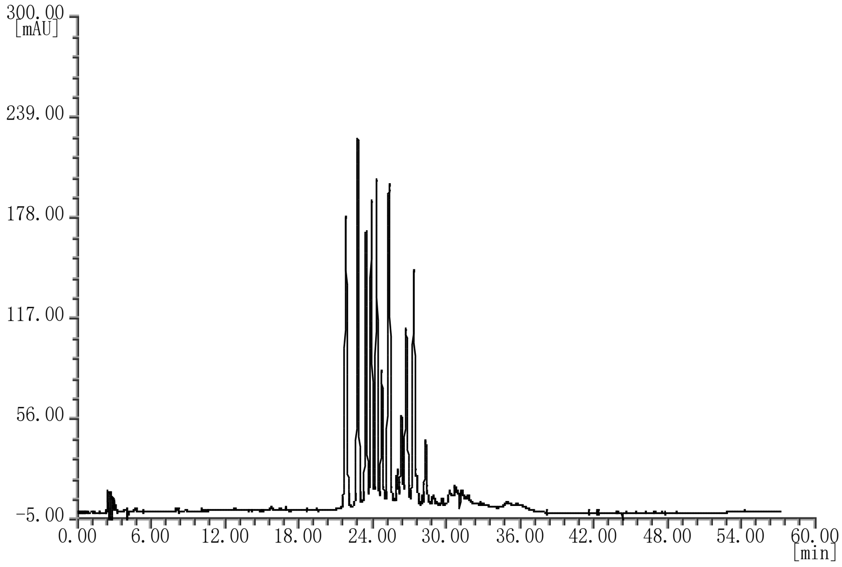

Figure. S2 Chromatogram under elution program 2.

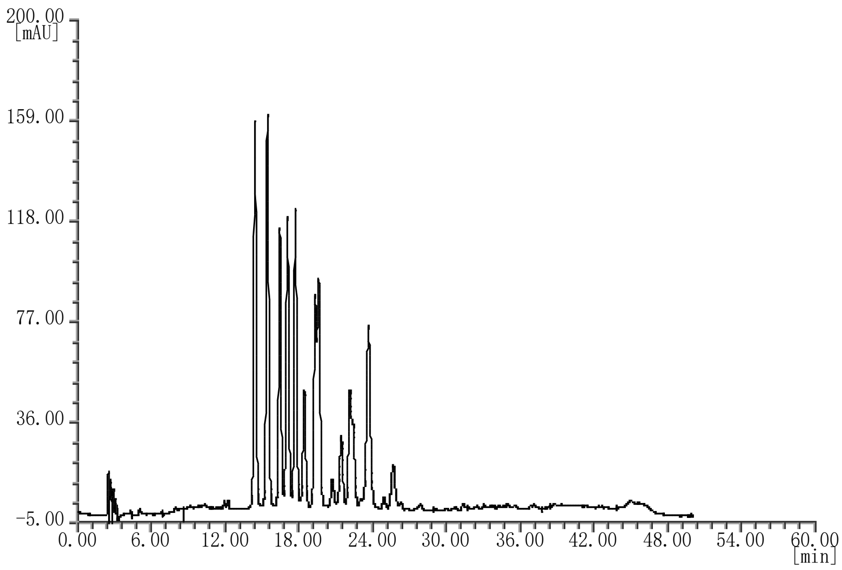

Figure. S3 Chromatogram under elution program 3.

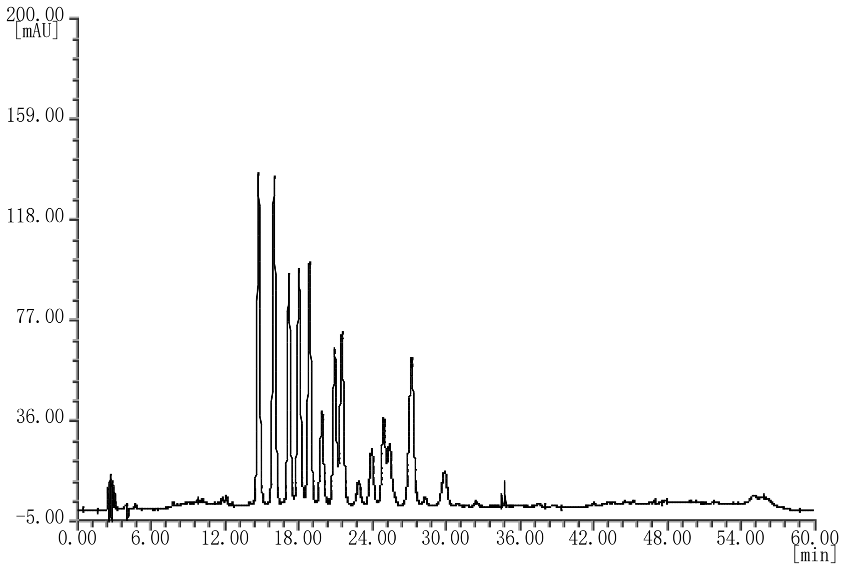

Figure. S4 Chromatogram under elution program 4.

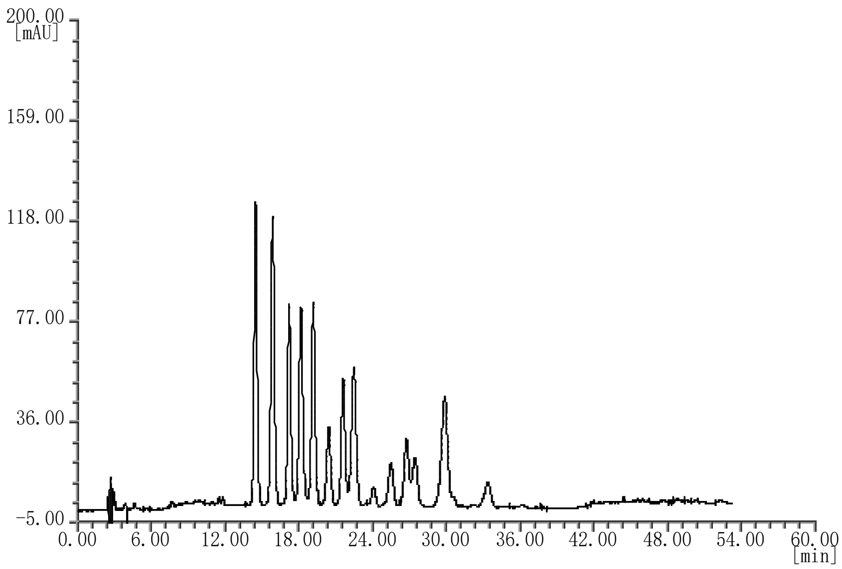

Figure. S5 Chromatogram under elution program 5.

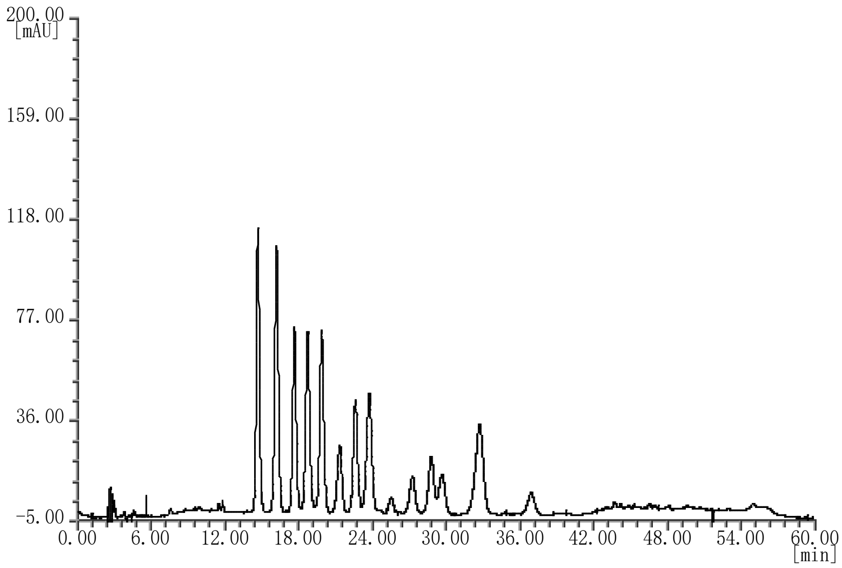

Figure. S6 Chromatogram under elution program 6.

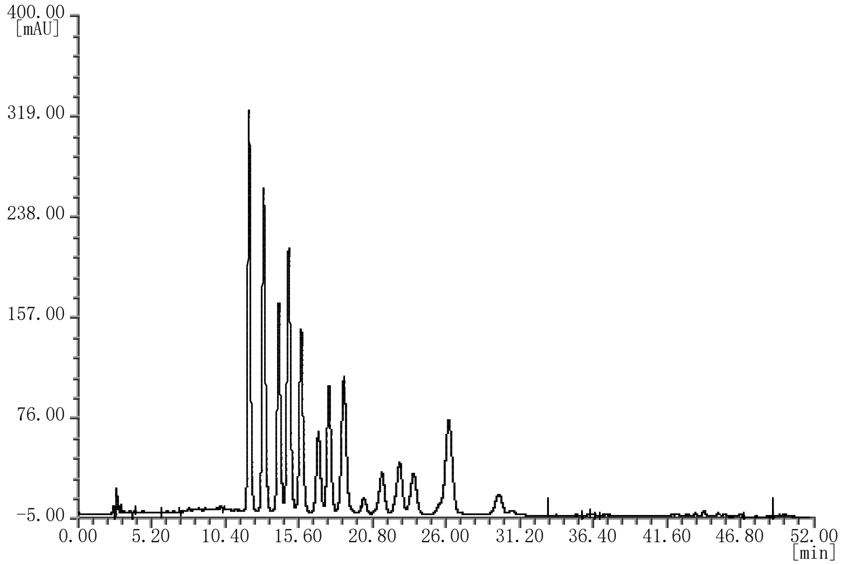

Figure. S7 HPLC chromatogram of the bilberry extract sample A1.

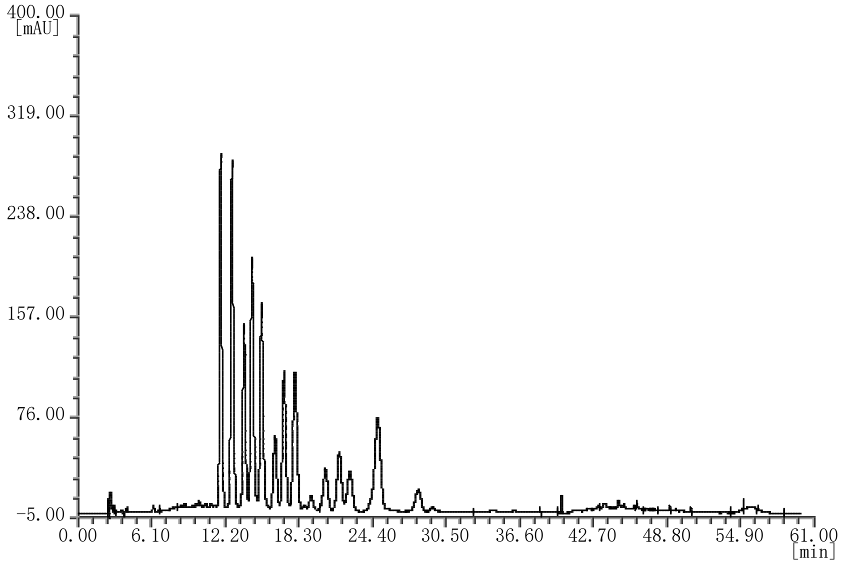

Figure. S8 HPLC chromatogram of the bilberry extract sample A2.

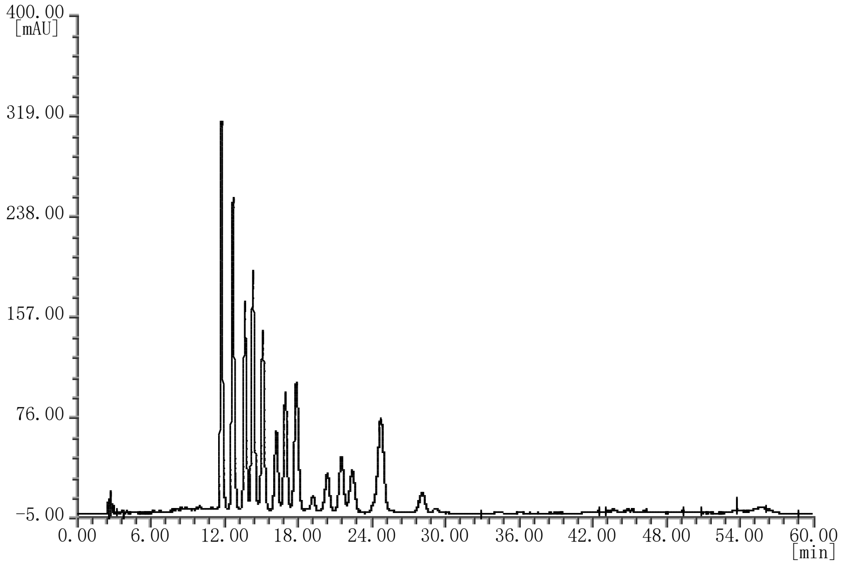

Figure. S9 HPLC chromatogram of the bilberry extract sample A3.

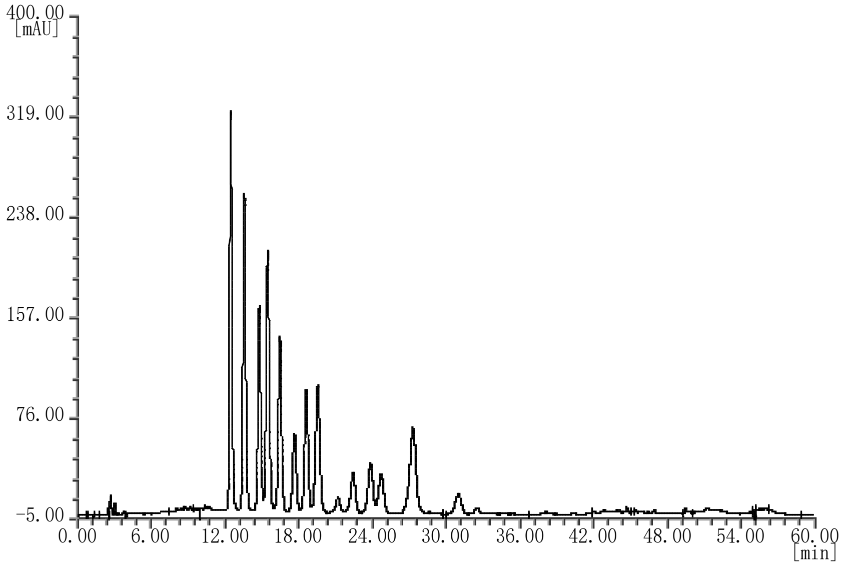

Figure. S10 HPLC chromatogram of the bilberry extract sample A4.

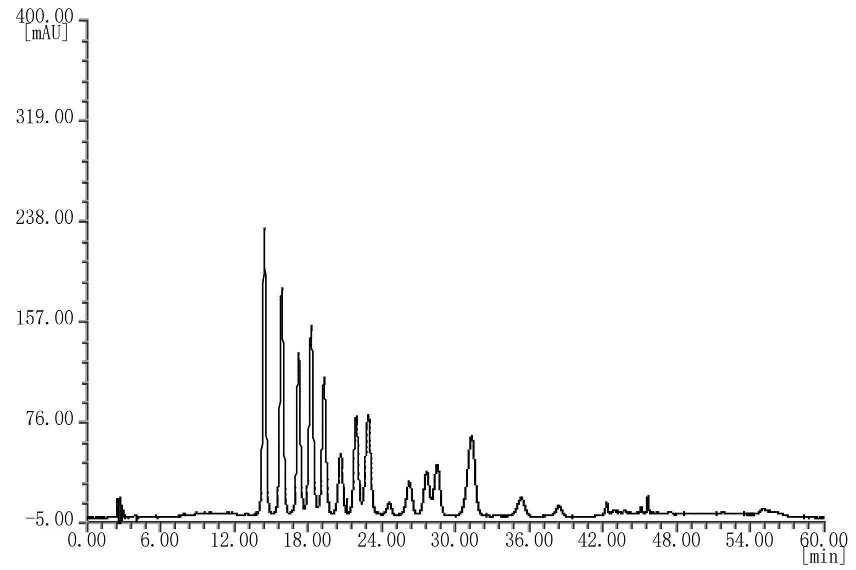

Figure. S11 HPLC chromatogram of the bilberry extract sample A5.

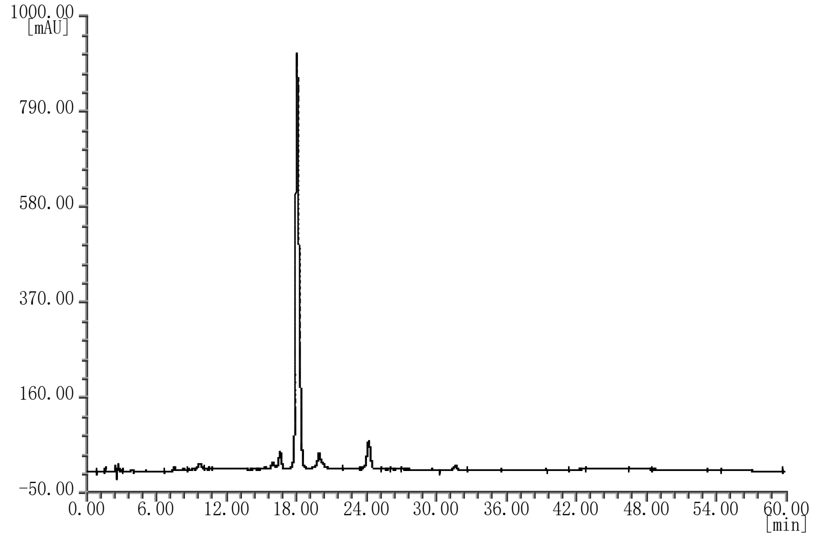

Fig. S12 HPLC chromatogram of the bilberry extract sample A6.

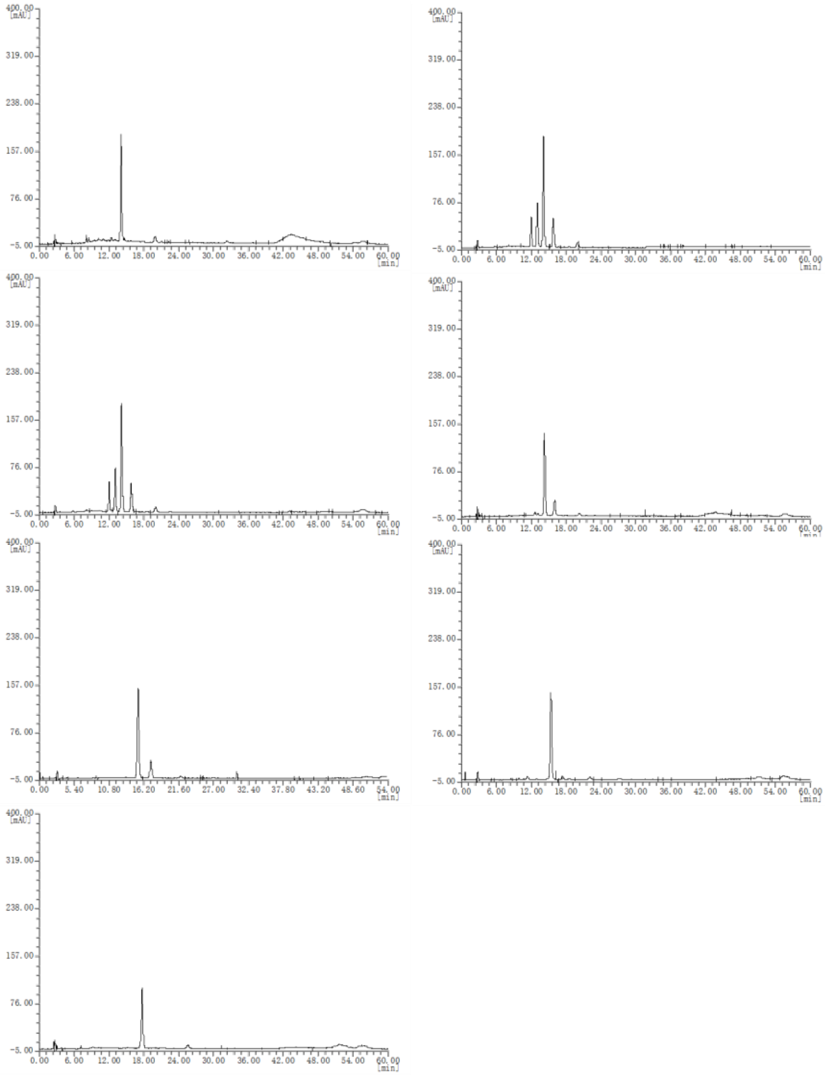

Fig. S13 HPLC chromatogram of the blueberry extract sample B1-B7.

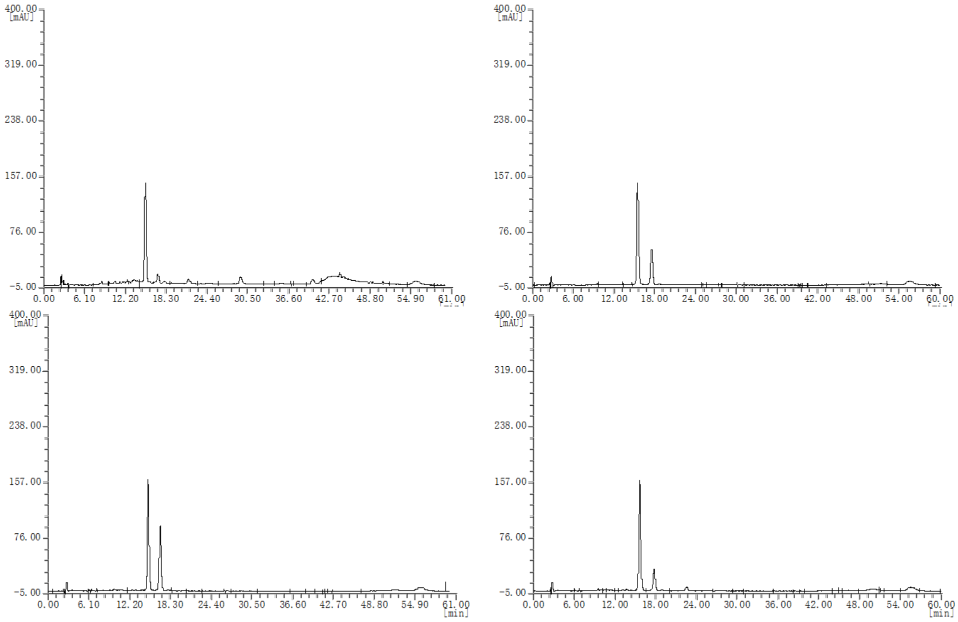

Fig. S14 HPLC chromatogram of the mulberry extract sample C1-C4.

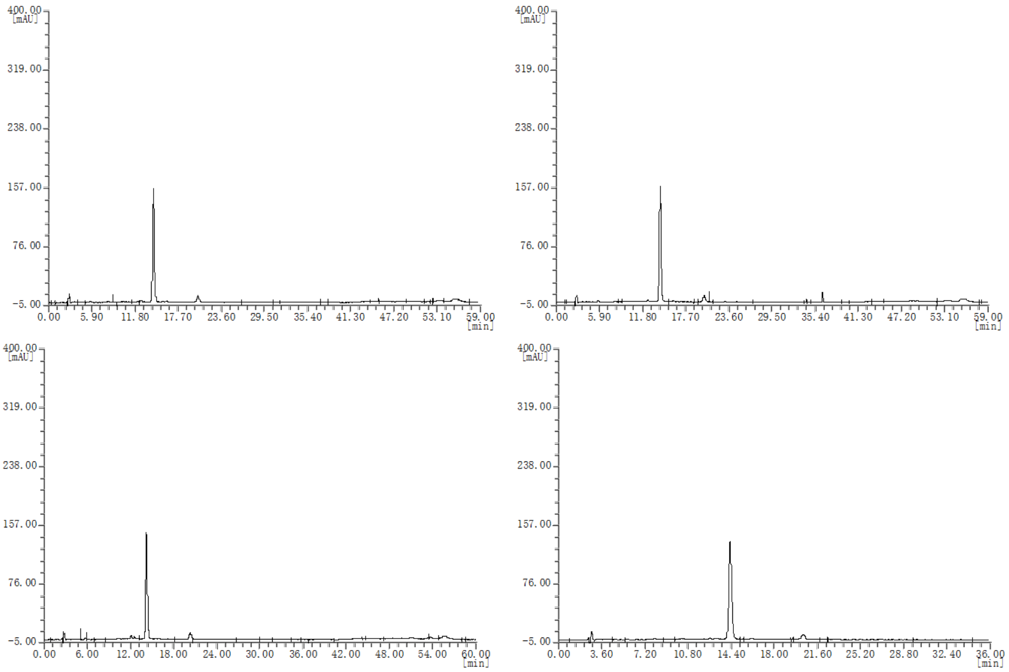

Fig. S15 HPLC chromatogram of the black rice berry extract sample E1-E4.
